# Carbimazole-induced Anaphylactic Shock: A Case Report

**DOI:** 10.5005/jp-journals-10071-23224

**Published:** 2019-08

**Authors:** Niraj Kumar Keyal, Sumal Thapa, Manoj Kumar Yadav

**Affiliations:** 1,3 Department of Critical Care and Emergency Medicine, B&C Medical College Teaching Hospital and Research Centre, Jhapa, Nepal; 2 Department of Anesthesia and Critical Care, B&C Medical College Teaching Hospital and Research Centre, Jhapa, Nepal

**Keywords:** Anaphylactic shock, carbimazole, hyperthyroidism

## Abstract

Carbimazole is an antithyroid drug that rarely presents as anaphylactic shock. We hereby report a case of 40-year-old female who was treated with carbimazole for thyrotoxicosis. Patient developed sweating, shortness of breath and altered sensorium after taking single dose of carbimazole. Patient was treated for anaphylactic shock. From this, we want to emphasize that carbimazole can rarely present as anaphylactic shock without any other allergic manifestation. So, patient should be counselled about this rare side effect and early approach to health care facility.

**Key messages:**

Patient on carbimazole should be aware of all side effects including rare side effects like anaphylactic shock. Studies are required to identify risk factors and new drugs for carbimazole allergy patients. Patient with thyrotoxicosis should also be screened for autoimmune thyrotoxicosis.

**How to cite this article:**

Keyal NK, Thapa S, Yadav MK. Carbimazole-induced Anaphylactic Shock: A Case Report. Indian J Crit Care Med 2019;23(8):380–381.

## INTRODUCTION

Carbimazole is a pro antithyroid drug that belongs to thioamide group. It inhibits peroxidase enzyme and affects thyroid hormone synthesis. It is preferred over propylthiouracil due to its less side effect like hepatotoxicity.^1^ It rarely causes anaphylaxis. There is no study that has shown that carbimazole can presents as anaphylactic shock. This is first case report of carbimazole presenting as anaphylactic shock.

## CASE DESCRIPTION

A 40-year-old female, without any significant past medical, allergic and surgical history presented to medicine outpatient department with history of loss of weight of 10 kilogram in two month, tremor, multiple episode of loose stool and irregular menstruation. She was diagnosed as hyperthyroidism and started on carbimazole. She again presented to the emergency department with sudden onset of shortness of breath, loss of consciousness, palpitation and impending doom after 1 hour of ingestion of carbimazole. Her vitals on admission were Glasgow coma scale (GCS) was 7/15, pulse rate was 140 beats/min, blood pressure was 70/40 mm Hg, respiratory rate was 40 breaths/min, oxygen saturation was 80% at 15 liters oxygen/min. On auscultation of chest, bilateral crepitation and wheeze were present. Cardiovascular, per abdominal examinations and skin examination were normal. Blood investigation showed leucocytosis. Liver function test, kidney function test was normal. Immediately patient was resuscitated and intubated. Patient was managed with intravenous fluid, adrenaline, hydrocortisone, chlorpheniramine and nor-adrenaline. Patient was extubated next day. Propylthiouracil was started for hyperthyroidism. Patient was shifted from intensive care unit on third day and was discharged on propylthiouracil. Patient was followed-up after one week and after that she had no further similar complaints.

## DISCUSSION

Carbimazole is preferred for treatment of thyrotoxicosis and is well tolerated but can produce adverse effects in 5% of patients.^2^ The major adverse effects are neutropenia, hepatotoxicity, agranulocytosis and skin rash.^3^ It rarely causes pleural effusion, lupus and vasculitis.^4–6^ Our patient did not develop any other allergic manifestation like skin rash, urticaria but presented as anaphylactic shock. There is no study and case reports that has shown the incidence of carbimazole induced anaphylactic shock.

Drugs are most common cause of anaphylaxis.^7^ Patient exposed to azole group of drugs is allergic to carbimazole. But, our patient did not have any such history. So, studies are required to identify the risk factors for carbimazole allergy.

Patient with autoimmune thyrotoxicosis and on carbimazole can develop angioedema and urticaria.^8^ But we did not ordered any test for autoimmune thyrotoxicosis as this was not available at our center. Propylthiouracil^8^ was used in our patient though there is cross reactivity of 50% between carbimazole and propylthiouracil. Patient should be prescribed alternative drug like propylthiouracil, potassium iodide and other drugs for thyrotoxicosis when developed allergic reaction to carbimazole.

To conclude studies are required to identify risk factor for group of patient that can develop carbimazole allergy. Patient with thyrotoxicosis should be screened for autoimmune thyrotoxicosis and should contact nearest healthcare facility if patient develop any drug side effects and prescribed alternative drugs.
